# Infectivity and pathogenicity of different hepatitis E virus genotypes/subtypes in rabbit model

**DOI:** 10.1080/22221751.2020.1858178

**Published:** 2020-12-24

**Authors:** Shuangshuang Li, Qiyu He, Li Yan, Manyu Li, Zhaochao Liang, Jingyi Shu, Fan Zhang, Ling Wang, Lin Wang

**Affiliations:** a Department of Microbiology and Infectious Disease Centre, School of Basic Medical Sciences, Peking University Health Science Centre, Beijing, People’s Republic of China; b Department of Severe Hepatology, Shanghai Public Health Clinical Centre, Fudan University, Shanghai, People’s Republic of China

**Keywords:** Hepatitis E virus, susceptibility, infectivity, pathogenicity, rabbit model

## Abstract

The pathogenicity of each hepatitis E virus (HEV) genotypes/subtypes may be different. This study aimed to investigate the infectivity and pathogenicity of different HEV genotypes/subtypes from different mammalian sources especially human in rabbits, and to assess whether rabbits are an appropriate animal model to study different HEV genotypes/subtypes. Thirty-seven rabbits were randomly divided into nine groups and inoculated with eight different HEV strains, including human-derived HEV3b (hHEV-3b), hHEV-4a, hHEV-4d and hHEV-4h, swine-derived HEV4d (sHEV-4d) and sHEV-4h, rabbit-derived HEV3 (HEV-3ra) and camel-derived HEV8. HEV RNA, antigen, anti-HEV and alanine aminotransferase (ALT) in serum or/and feces were monitored weekly. One rabbit from each group was euthanized at seven weeks post inoculation and the liver specimens were taken for histopathological analysis and immunofluorescence staining of HEV ORF2 proteins. hHEV-4d, sHEV-4d and HEV-3ra infections were successfully established in rabbits and typical acute hepatitis symptoms were observed, including viraemia/antigenemia, fecal virus/antigen shedding, elevated ALT level and liver histopathological changes. One rabbit infected with HEV-3ra showed chronic infection. hHEV-4d and sHEV-4d are less infectious and pathogenic than HEV-3ra in rabbits. hHEV-3b and HEV8 only caused inapparent infection in rabbits as 60% (3/5) and 20% (1/5) of the rabbits seroconverted to anti-HEV, respectively. No obvious signs of HEV infection in rabbits inoculated with hHEV-4a, hHEV-4h and sHEV-4h. The infectivity and pathogenicity of different HEV genotypes/subtypes in rabbits is different, which may be related to the species specificity of HEV. Rabbit can be used as an animal model for the study of HEV-3ra and more importantly human HEV-4d.

## Introduction

Hepatitis E virus (HEV), mainly transmitted via fecal-oral route, is a single-stranded positive-sense RNA virus with a genome of about 7.2 kb in length and three open reading frames (ORF) flanked by two untranslated regions at the 5′ and 3′ end [1,2]. HEV infection usually presents as acute and self-limiting hepatitis and the course of the disease is generally 4–6 weeks. Patients may suffer from fatigue, loss of appetite, nausea and elevated serum ALT levels [1,2]. However, fulminant hepatic failure induced by HEV infection can occur during pregnancy, with a mortality rate up to 20% [3,4]. Among immunocompromised individuals, such as solid organ transplantation recipients and HIV/AIDS patients, HEV infection can lead to chronic hepatitis and progress to liver fibrosis and even cirrhosis [5–7]. In addition, a variety of extra-hepatic symptoms, including neurological symptoms and kidney injury, were reported related to HEV infection [8–10].

HEV belongs to the family *Hepeviridae*, which includes five species (*Orthohepevirus* A–D and *Piscihepevirus*). Species *Orthohepevirus* A is closely related to infections in humans and consists of at least 8 genotypes (HEV1-8) [1,2]. The majority of human infections are caused by HEV1-4. HEV1 and HEV2 only infect humans causing large-scale outbreaks and epidemics via contaminated water in developing regions with poor sanitation. HEV3 and HEV4 are zoonotic and mainly induce sporadic cases in developed countries and China through consumption of meat products from infected animals especially pigs [1,2]. In 2009, a novel HEV strain was isolated from the farmed rabbits in China [11] and was currently classified as genotype 3 (HEV-3ra) due to its highest nucleotide sequence identity with HEV3 [12]. Later studies reported cases of HEV-3ra infecting humans and causing both acute and chronic hepatitis E [13,14]. HEV5 and HEV6 were isolated from wild boars in Japan, but they have not been found to infect humans [15,16]. HEV7 was isolated from dromedary camels in 2014 [17] and subsequently was found causing chronic hepatitis E in a patient, confirming that HEV7 is zoonotic [18]. In 2016, HEV8 was isolated from Bactrian camels in China [19], and could successfully infect cynomolgus monkeys, implying risks of infecting humans [20]. In addition, a number of recent studies have also found that rat HEV, which is genetically distant from HEV1-8 and belongs to species *Orthohepevirus* C, can also infect humans, drawing extensive attentions [21,22]. Therefore, as the animal reservoirs of HEV continue to expand, the risks of HEV exposure and cross-species transmission are getting higher.

The infectivity and pathogenicity of each HEV genotypes/subtypes may be different. Therefore, it is essential to develop an appropriate animal model to evaluate the infectivity and pathogenicity of different HEV genotypes. Commonly used animal models for HEV research include non-human primates, pigs, humanized mice and rabbits [2]. The aforementioned animal models for HEV infection were commonly established by the administration of feces-derived inocula [23,24] as feces are the main transmission vehicle of HEV [1]. Non-human primates are susceptible to HEV1-4, 5, 7 and 8, and the infected animals can manifest with acute hepatitis [20,24–26]. As the natural reservoir of HEV3 and HEV4, pigs are susceptible to these two genotypes, but no obvious symptoms are observed during the infection [27]. Humanized mice are a novel animal model for HEV1 and HEV3 infection, but the infectivity of HEV4 has not been investigated in this model [2,28,29]. Among all the existing animal models, HEV4 infection is less studied than that of HEV1 and HEV3 [23], while HEV4 is the dominated genotype circulating in China and emerging in Europe recently [1,2]. Rabbits infected with HEV-3ra have obvious symptoms and signs such as fecal virus shedding, viraemia, seroconversion to anti-HEV, elevated ALT levels, liver inflammation and pathological changes [30]. The relatively small size, low cost, simpler experimental procedure and less ethical issues make it a promising small animal model for large-scale HEV infection study. However, the breadth of susceptibility of rabbit to other zoonotic HEV genotypes/subtypes warrants further investigation especially the human-pathogenic HEV3 and HEV4 strains, and the newly discovered HEV8 in Chinese Bactrian camels. In this study, eight HEV strains of different genotypes/subtypes especially HEV4 from different mammalian sources were used to infect rabbits to explore whether rabbits can be used as animal models for the study of different HEV genotypes/subtypes and to compare the infectivity and pathogenicity of these HEV strains in rabbits.

## Materials and methods

### Virus inoculum

In this study, a total of eight different HEV strains of genotypes/subtypes obtained from different mammalian sources were used and the details of the eight strains are listed in Table 1. The HEV-positive fecal samples were diluted in sterile phosphate buffer saline, respectively, to make 10% (wt/vol) suspension and then centrifuged at 4000× *g* at 4°C for 30 min. The clarified suspension was filtered through 0.45 and 0.22 μm filters and titrated subsequently. All eight HEV-positive inocula were adjusted to 1 × 10^6^ copies/mL. The negative inoculum was prepared from fecal sample of a non-infected rabbit.

**Table 1.** Hepatitis E virus strains used in the study.

Group	Animal number	Genotype/subtype	Origin of inoculum^a^	Infectivity^b^	GenBank number
A	5	hHEV-3b	Chronic hepatitis E patient	Pregnant rabbit [32]	MF996356
B	5	hHEV-4d	Acute hepatitis E patient	NT	MT993748
C	5	sHEV-4d	Farmed pig	Pregnant rabbit [32]	MT993749
D	5	sHEV-4h	Farmed pig	NT	MT993750
E	5	HEV8	Bactrian camel	Cynomolgus monkey [20]	MH410174
F	5	HEV-3ra	Farmed rabbit from Beijing	Cynomolgus monkey [31], rabbit [30]	JX109834
G^c^	2	hHEV-4a	Acute hepatitis E patient	NT	KP325707
H^c^	2	hHEV-4h	Acute hepatitis E patient	NT	KP325697
I	3	negative control	Farmed rabbit	NA	–

^a^ All virus inocula were made from feces.

^b^ Animals successfully experimentally infected.

^c^ The original clinical samples were only enough for inoculation of two rabbits.

### Experimental design of animal study

Thirty-seven 3-month-old Japanese white rabbits weighing about 2–3 kg were randomly selected. Serum and fecal samples were collected weekly for two consecutive weeks to test alanine aminotransferase (ALT) for establishing the baselines. All samples were negative for anti-HEV antibodies by an enzyme-linked immunosorbent assay (ELISA) and HEV RNA by a previously described reverse transcription-nested polymerase chain reaction (RT-nPCR) [32].

Rabbits were randomly divided into nine groups. Rabbits in Group A-F (*n* = 5, each group) were inoculated intravenously with 1 mL of human-derived HEV-3b (hHEV-3b), human-derived HEV-4d (hHEV-4d), swine-derived HEV-4d (sHEV-4d), swine-derived HEV-4h (sHEV-4h), Bactrian camel-derived HEV8 and rabbit-derived HEV-3 (HEV-3ra) positive inoculum, respectively. The hHEV-4a and hHEV-4h strains were made from limited clinical samples and were not enough for the inoculation of five rabbits; therefore, Group G and H consist of two rabbits each. Group I rabbits (*n* = 3) were inoculated intravenously with 1 mL of negative inoculum as a negative control (Table 1). All rabbits were housed in separate cages with adequate water and food. The animal experiments were approved by the Committee of Laboratory Animal Welfare and Ethics, Peking University Health Science Centre.

### Samples collection and processing

Serum and fecal samples were collected weekly and stored at −80°C. Serum samples were tested for ALT, anti-HEV antibody, HEV antigen and HEV RNA through standard methods. Fecal samples were processed as previously described and tested for HEV antigen and HEV RNA. Seven weeks after inoculation, one rabbit in each group (except Group G and H) was euthanized and liver specimens were taken and fixed in neutral 4% paraformaldehyde for 3 days for histopathological analysis and immunofluorescence staining of HEV ORF2 proteins.

### Determination of ALT concentrations

The serum samples of rabbits were tested for serum ALT concentrations using standard methods on a Hitachi Automatic Clinical Analyzer 7180. Rabbits were considered to have liver injury when the serum ALT concentration exceeded the pre-challenge level by more than two-fold.

### Detection of anti-HEV, HEV antigen and HEV RNA

Anti-HEV antibody and HEV antigen were detected by commercial ELISA kits (Wantai, Beijing, China) according to the manufacturer’s instructions. Signal-to-cut-off (S/CO) values were calculated and values >1 were considered positive.

Total RNA was extracted from 100 μL of serum or fecal suspensions prepared as above using TRIzol reagent (Invitrogen, Burlington, ON, Canada) following a standard instruction. The RNA was then amplified by RT-nPCR for partial ORF2 genome on an automatic PCR system (Applied Biosystems ProFlex PCR, USA) and HEV viral load was determined using a commercial one-step real-time quantitative PCR assay (GoTaq® Probe 1-step RT-qPCR System Kit, Promega, Wisconsin, USA), as previously described [32].

### Histopathology and immunofluorescence staining

The liver tissue were fixed in 10% neutral buffered formalin and embedded in paraffin and cut into 5 μm serial sections. Slides were stained with haematoxylin and eosin (H&E). The samples were photographed and analysed under a microscope (Olympus CX31, Japan) equipped with a digital camera with the assistance of Motic Images Plus 2.0 software.

For immunofluorescence, tissue and organ sections were deparaffinized, hydrated, and water-bath heated for antigen retrieval. HEV proteins were visualized by using HEV ORF2-specific antibodies (Bioss, Woburn, USA). Nuclear staining was achieved with DAPI (Beyotime, Shanghai, China). Staining sections were photographed and analysed by fluorescence microscopy (Nikon DS-U3, Japan).

### Phylogenetic analysis

Nucleotide sequences of the eight strains, and other sixty-six ICTV proposed reference sequences obtained from GenBank, were aligned and analysed with MEGA X [33]. Phylogenetic trees were constructed by using the neighbour-joining method with bootstrap values calculated from 1000 trees. The evolutionary distances were computed using the Maximum Composite Likelihood method.

## Results

### Phylogenetic analysis of the different genotypes/subtypes HEV strains

**Figure 1.**
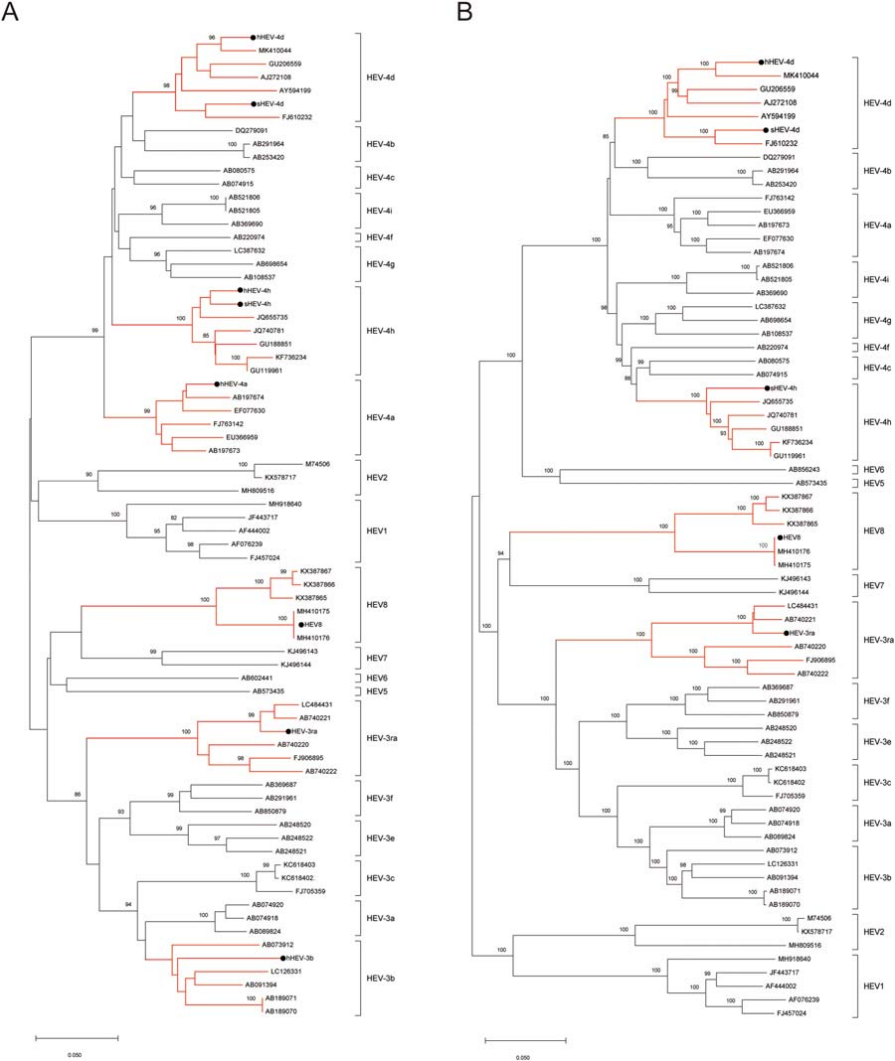
Phylogenetic analysis of the strains used to infect rabbits. (A) Phylogenetic analysis of the eight strains based on a partial nucleotide sequence about 350 bp of the ORF2 region. (B) Phylogenetic analysis of nearly complete sequences of five strains (HEV-3ra, hHEV-4d, sHEV-4d, sHEV-4h and HEV8). The complete or nearly complete sequences of the above five virus strains used in the study were obtained. Sixty-six ICTV proposed reference strains were used and the GenBank accession numbers of all reference sequences are shown in the figure. The phylogenetic trees were constructed by the neighbour-joining method. Numbers indicate nodes where bootstrap support was >80% out of 1000 replicates. Bar: nucleotide sequence distances; Dots: strains used to infect rabbits in the study. GenBank numbers: hHEV-3b, MF996356; hHEV-4a, KP325707; hHEV-4d, MT993748; hHEV-4h, KP325697; sHEV-4d, MT993749; sHEV-4h, MT993750; HEV-3ra, JX109834; HEV8, MH410174.

The phylogenetic tree was constructed by the neighbour-joining method based on a partial nucleotide sequence about 350 bp of the ORF2 region and involved seventy-four nucleotide sequences including the eight strains used in the current study and the ICTV proposed reference strains [33]. The phylogenetic tree shows that each of the eight strains is clustered with their related reference strains of the same genotypes/subtypes, respectively (Figure 1(A)). We also obtained the complete or nearly complete sequences of five virus strains used in the study (HEV-3ra, hHEV-4d, sHEV-4d, sHEV-4h and HEV8). The phylogenetic tree, constructed with the nearly complete sequences (about 7000 bp), also shows that these five strains were clustered with their related reference strains of the same genotypes/subtypes, respectively (Figure 1(B)).

### Different HEV genotypes/subtypes showed different infectivity in rabbits

Rabbits were inoculated intravenously with the eight HEV strains of different genotypes/subtypes from different mammalian sources (Table 1). The rabbits in Group A (hHEV3b) and E (HEV8) had no viraemia and fecal virus shedding during the study, while 60% (3/5) and 20% (1/5) of the rabbits, respectively seroconveted to anti-HEV, suggesting an inapparent infection. All rabbits in Group B (hHEV-4d) and F (HEV-3ra) showed acute HEV infection except one rabbit (1/5, 20%) from Group F showed chronic HEV infection with fecal virus shedding for 14 weeks. Four rabbits (4/5, 80%) in Group C (sHEV-4d) showed acute HEV infection. No sign of infection was observed in Group D (sHEV-4h), G (hHEV-4a), H (hHEV-4h) and I (negative control group) during the entire study (Table 2).

**Table 2.** Infectivity of different HEV genotypes/subtypes in rabbits.

Group	Genotype/subtype	Infectivity (%)			
		Acute infection^a^	Chronic infection^a^	Inapparent infection^a^	No sign of infection
A	hHEV-3b	0/5 (0)	0/5 (0)	3/5 (60)	2/5 (40)
B	hHEV-4d	5/5 (100)	0/5 (0)	0/5 (0)	0/5 (0)
C	sHEV-4d	4/5 (80)	0/5 (0)	0/5 (0)	1/5 (20)
D	sHEV-4h	0/5 (0)	0/5 (0)	0/5 (0)	5/5 (100)
E	HEV8	0/5 (0)	0/5 (0)	1/5 (20)	4/5 (80)
F	HEV-3ra	4/5 (80)	1/5 (20)	0/5 (0)	0/5 (0)
G	hHEV-4a	0/2 (0)	0/2 (0)	0/2 (0)	2/2 (100)
H	hHEV-4h	0/2 (0)	0/2 (0)	0/2 (0)	2/2 (100)
I	Negative control	0/3 (0)	0/3 (0)	0/3 (0)	3/3 (100)

NA, not applicable; NT, not tested.

^a^ Acute infection: fecal virus shedding less than 3 months; chronic infection: fecal virus shedding over 3 months; inapparent infection: no signs of HEV infection except for seroconversion to anti-HEV antibody.

**Figure 2.**
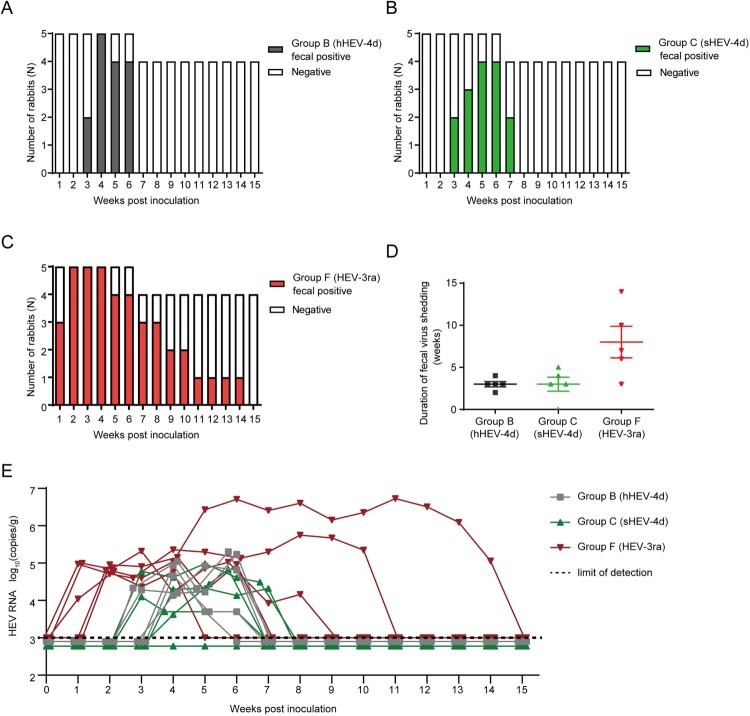
Duration of fecal virus shedding and fecal viral load in Group B, C and F. (A–D) Duration of fecal virus shedding determined by RT-nPCR results among group B, C and F. (E) Fecal viral load quantified by RT-qPCR results among group B, C and F. Limit of detection showed in dotted line.

All five rabbits in Group B were successfully infected with hHEV-4d. Fecal virus shedding was first observed at 3 wpi in two rabbits and at 4 wpi in the other three. All rabbits stopped fecal virus shedding before 7 wpi and the duration of fecal virus shedding was between 2 and 4 weeks (Figure 2(A)). In Group C, four rabbits were successfully infected with sHEV-4d and rabbits started fecal virus shedding varied from 3 wpi to 5 wpi. All rabbits stopped fecal virus shedding before 8 wpi and the duration of fecal virus shedding was between 3 and 5 weeks (Figure 2(B)). Our previous study has demonstrated that HEV-3ra is infectious in rabbits [30]. Rabbits in Group F were infected with HEV-3ra and served as a positive control. All rabbits started fecal virus shedding within 2 wpi and the duration of fecal virus shedding was between 3 and 14 weeks (Figure 2(C)). The fecal virus shedding lasted longer in HEV-3ra-infected rabbits than that in human- or swine-derived HEV-4d-infected rabbits (Figure 2(D)). The fecal viral load of rabbits in Group F (10^5^–10^7^ copies/g) was higher than that in Group B and C (10^3^–10^5^ copies/g, Figure 2(E)). In addition, except 20% (1/5) of Group B rabbits and 60% (3/5) of Group F rabbits developed transient viraemia, none of the other rabbits showed viraemia during the entire study.

**Figure 3.**
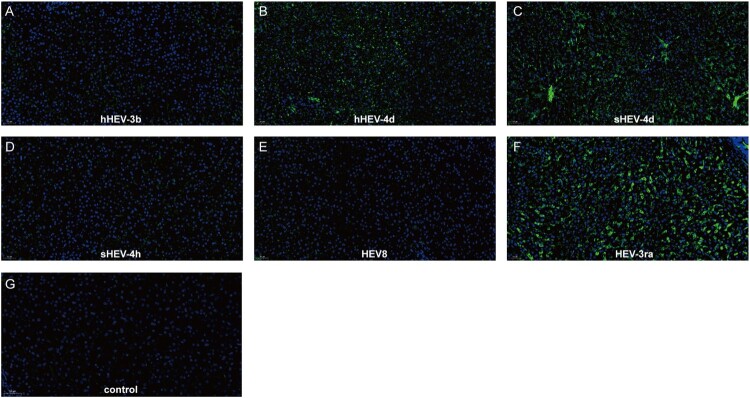
Immunofluorescence staining of HEV ORF2 in liver tissues. Positive signals of ORF2 were observed in liver section in successfully infected group B, C and F. No obvious positive signals were observed in group A, D, E and Control group I. Original magnification, ×20; HEV ORF2 antigen was stained with HEV ORF2-specific antibody in green; nuclei were stained with DAPI in blue.

Immunofluorescence results of ORF2 in the liver were in accordance with the results of fecal virus shedding. Positive signals of ORF2 were observed in infected rabbits from Group B, C and F, while no obvious positive signals were seen in Groups A, D, E and negative control Group I (Figure 3). Due to the limited animal size, no rabbit was euthanized in Group G and H.

### Kinetics of HEV antigen levels in rabbits of different groups

**Figure 4.**
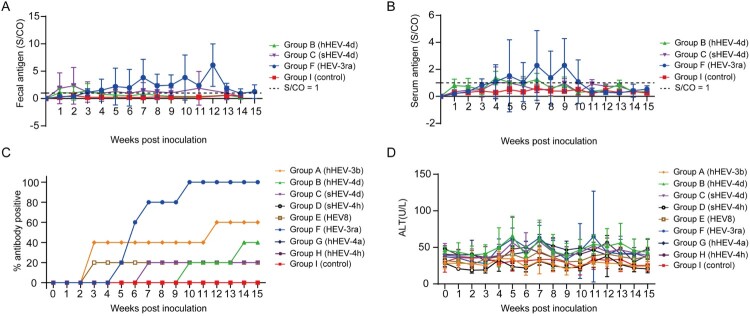
Kinetics of fecal antigen, serum antigen, anti-HEV antibodies and alanine aminotransferase (ALT) levels. (A) Kinetics of fecal antigen in successfully infected rabbits of group B, C and F. (B) Kinetics of serum antigen in successfully infected rabbits of group B, C and F. (C) Dynamic anti-HEV antibody positivity in group A–I. S/CO, signal/cut-off, S/CO = 1 showed in dotted line, S/CO > 1 was positive (D) Dynamic ALT changes in group A–I.

Fecal and serum HEV antigen were monitored weekly. Fecal antigen was first detected in Group B rabbits at 1 wpi with low S/CO values between 1 and 2 and turned negative at 4 wpi. In Group C, fecal antigen was also first detected at 1wpi with slightly higher S/CO values of 1–4 than that in Group B. The S/CO values decreased at 3 wpi and stayed around 1–2 until 13 wpi. Fecal antigen was detected from 3 wpi to 14 wpi in Group F rabbits and S/CO values were at a relatively high level (Figure 4(A)). Results of serum antigen detection showed that rabbits in Group B were positive from 4 wpi to 7 wpi. Rabbits in Group C showed transient serum positivity for HEV antigen at 4 wpi. In Group F, serum antigen was positive from 4 wpi to 11 wpi. Both fecal and serum antigen stayed negative in Group A, D, E, G, H and negative control Group I (Figure 4(B)).

### Seroconversion for anti-HEV antibody in rabbits of different groups

In Group A and E, rabbits showed no symptoms of infection or pathological changes in the liver, but 60% (3/5) and 20% (1/5) of rabbits were seroconverted to anti-HEV antibodies, respectively, which suggested an inapparent infection. In Group B, each rabbit was seroconverted for anti-HEV antibodies at 10 and 14 wpi, respectively, while the remaining rabbits stayed seronegative during the study. Only one rabbit in Group C showed seroconversion for anti-HEV antibodies at 7 wpi. All rabbits in Group F were seroconverted for anti-HEV antibodies. The rabbits in Group D, G, H and I did not show seroconversion during the entire study (Figure 4(C)).

### Different HEV genotypes/subtypes showed different pathogenicity in rabbits

ALT levels of the rabbits were dynamically monitored weekly. The ALT levels of rabbits in Group B and C started to increase at 4 and 5 wpi, respectively with three rabbits from each groups increased more than twice the baseline and both recovered at 9 wpi. Group F showed elevated ALT levels at 4 wpi in which four rabbits showed elevated ALT levels more than 2 times of the baseline and recovered at 12 wpi. The ALT levels of rabbits in other groups (Group A, D, E, G and H) and the negative control group (Group I) did not increase significantly during the study (Figure 4(D)).

**Figure 5.**
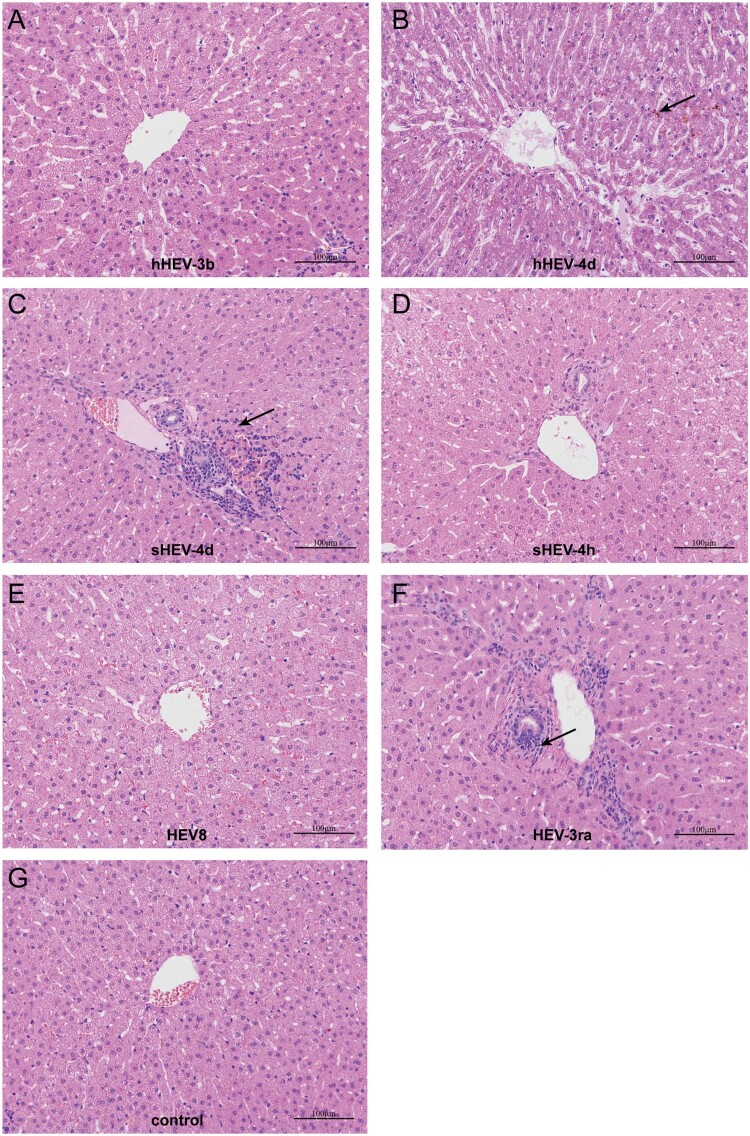
Histopathology of liver tissues. Disordered structure of liver tissues and infiltration of inflammatory cells were observed in liver sections of infected rabbits in group B, C and F. Mild cholestasis was also observed in group B. No obvious pathological changes were seen in group A, D, E and negative group I. Arrows indicate infiltration of inflammatory cells and cholestasis. Original magnification, ×10.

In order to assess the pathogenicity in the liver, one rabbit in each group (except for G and H) was euthanized at 7 wpi. H&E staining showed the disordered structure of liver tissues and infiltration of inflammatory cells in liver sections in Group B, C and F. Mild cholestasis was also observed in Group B. No obvious pathological changes were seen in Groups A, D, E and negative control Group I (Figure 5).

## Discussion

The current study investigated the infectivity and pathogenicity of eight different HEV genotypes/strains in rabbits, including human-derived HEV-3b, HEV-4a, HEV-4d and HEV-4h, swine-derived HEV-4d and HEV-4h, rabbit-derived HEV3 and camel-derived HEV8, providing valuable information of the susceptibility and future application of the rabbit model in HEV study especially information about human HEV4 strains. These isolated strains are representative strains of the HEV genotypes/subtypes circulating in China. Although HEV3 is prevalent in many developed countries, it is not the dominant genotype in China [1,34]. There were few reports of HEV3 sequences in human in China and all isolated strains so far belong to subtype HEV-3b, so the human-derived HEV-3b we used is representative of this genotype/subtype in China [34]. Hepatitis E in China is mainly caused by HEV4 with diverse subtypes and the five HEV4 strains we used represent three major HEV4 subtypes circulating in China [35–37]. HEV8 strains were recently discovered in Chinese Bactrian camels [19,20]. To our knowledge, there are so far only four complete genomes of camel-derived HEV8 strains reported worldwide, including the strain we used in the current study [19,20].

Among the existing animal models for HEV infection, nonhuman primates, pigs, rabbits and human liver chimeric mice are the most frequently used ones [2]. Each model possessed unique advantages and potential limitations. The most desirable animal models should resemble high clinical relevance and is susceptible to human HEV strains. Rabbit can be readily infected with zoonotic HEV-3ra and can present with features of acute or chronic-type HEV infection similar to patients [13,14,30]. Although HEV-3ra belongs to HEV3, previous attempts of infecting rabbits with human HEV3 had been unsuccessful [30,38]. Two recent studies found that a wild boar HEV3 and a swine HEV3 strain can infect rabbits, but the infection efficiency is rather low with only transient fecal virus shedding in most of the rabbits [39,40]. In the present study, rabbits inoculated with hHEV-3 showed no detectable viraemia/fecal virus shedding, increase of ALT level and histopathological changes in the liver. However, 60% (3/5) of the inoculated rabbits seroconverted to anti-HEV at the end of the study indicating inapparent infection. We previously found that this hHEV-3 strain could induce robust infection in pregnant rabbits [32]. This observation may be explained by the different host immune status during pregnancy, which increased the host susceptibility to certain pathogens and increased disease severity [41]. The underlying mechanism specific to HEV warrants future study. Overall, such results along with previous studies suggest that rabbit is probably not naturally susceptible to human/swine HEV3.

HEV4 is the dominant genotype in China and is prevalent in many other Asia countries including Japan, South Korea and Singapore [1]. Recently, HEV4 has also been isolated from European countries and sometimes cause small outbreaks [42]. Meanwhile, several studies reported that chronic HEV infection and HEV-associated neurological injury can be induced by HEV4 [43,44]. Several animal models were reported susceptible to HEV4 such as nonhuman primate, pigs, rabbits and gerbils, but most of the HEV4 strains used were isolated from swine [23,24]. Infection of human HEV4 strains in small animal model is less studied than HEV1 and HEV3 [28,29]. The result of the present study showed that rabbit is susceptible to HEV4 strains including human isolate. Only subtype HEV-4d can induce successful infection in rabbits suggesting that a potential discrepancy of infectivity of different HEV4 subtypes. Rabbits infected with both human and swine HEV-4d manifest as acute hepatitis E with elevated ALT levels and liver pathology. In general, HEV-3ra strain induced more robust infection in rabbits with longer duration of fecal virus shedding, antigenemia and higher viral load in feces than both human and swine-derived HEV-4d. Human- and swine-derived HEV-4d had similar infectivity and pathogenicity in rabbits. The successful establishment of human HEV-4d infection in rabbits renders more clinical relevance to this model. Infection of rabbits with camelid HEV8 strain was not successful, only one rabbit showed seroconversion to anti-HEV at the end of the study. More animal species and sample size should be used in future study to further investigate the cross-species ability and pathogenicity of HEV8.

In summary, the present study demonstrated that different HEV genotypes/subtypes showed different infectivity and pathogenicity in rabbits, which may be related to the species specificity of HEV. Rabbit can be used as an animal model for the study of rabbit-derived HEV3 and more importantly, human HEV4. Further study using more HEV isolates of different genotypes/subtypes is warranted.
